# JKAP relates to disease risk, severity, and Th1 and Th17 differentiation in Parkinson's disease

**DOI:** 10.1002/acn3.51420

**Published:** 2021-07-21

**Authors:** Qingwei Yang, Jingcong Zhuang, Pingping Cai, Longling Li, Rong Wang, Zhongjie Chen

**Affiliations:** ^1^ Department of Neurology Zhongshan Hospital Xiamen University Xiamen China; ^2^ School of Medicine Xiamen University Xiamen China; ^3^ Department of Neurology Fujian Medical University Xiamen Humanity Hospital Xiamen China

## Abstract

**Objective:**

JNK pathway‐associated phosphatase (JKAP) is previously reported to regulate immune/inflammatory process via T‐cell signaling, and closely involves in neurological diseases, while its implication in Parkinson's disease (PD) is unknown. Therefore, this study aimed to investigate the correlation of JKAP with Th1/Th2/Th17 cells and their clinical roles in PD patients, and then further explore the effect of JKAP on regulating CD4^+^ T‐cell differentiation in PD.

**Methods:**

Totally 50 PD patients and 50 age‐/gender‐matched controls were enrolled. Their blood samples were collected and proposed to ELISA and flow cytometry assays for JKAP, Th1, Th2, and Th17 measurements. In vitro, CD4^+^ T cells were isolated from PD patients then transfected with JKAP overexpression and knockdown *Lentivirus*, followed by detection of markers (CD25^+^ cell proportion, CD69^+^ cell proportion, IFN‐γ, IL10, and IL17).

**Results:**

JKAP was downregulated in PD patients compared to controls, which also showed good potency to discriminate them. Besides, JKAP negatively correlated with Th1 and Th17 cell proportions, but did not associate with Th2 cell proportion in PD patients; Interestingly, JKAP did not correlated with Th1, Th2, or Th17 cell proportions in controls. Furthermore, JKAP correlated with some parts of unified Parkinson’s Disease Rating Scale (UPDRS) and Mini‐Mental State Examination (MMSE) score. In vitro, JKAP overexpression repressed CD4^+^ T‐cell activation and its differentiation into Th1 and Th17 cells in PD, while JKAP knockdown appeared opposite effect.

**Interpretation:**

JKAP associates with disease risk and severity, correlates with Th1 and Th17 cells, and regulates CD4^+^ T‐cell activation/differentiation in PD.

## Introduction

Parkinson's disease (PD) is a common neurological disorder which affects 315 (95% 113–873) subjects per 100,000 population globally, and 79.5 to 193.3 subjects per 100,000 population globally in China.^1^, ^2^ Of note, along with the disease course, PD patients not only suffer from motor symptoms, but also stand non‐motor symptoms and complications, making PD a great health issue over the world.^3^ Although it is considered that genetic and environmental factors attribute to its development,^4^ up to now, 90 independent risk‐associated gene variants of PD (or mutations) have been discovered along with the advancement of genome‐wide sequencing; meanwhile, 11 independent environmental factors related to PD such as pesticide exposure, beta‐blocker use are discovered.^3^, ^5^ However, the detailed etiology of PD is still tenebrous; therefore, the effort to discover PD pathology and markers is never stopped.

JNK pathway‐associated phosphatase (JKAP), also named as dual specificity protein phosphatase 22 (DUSP22), is reported to be involved in the regulation of immunity and inflammation; meanwhile, it is also implicated in the development and progression of neurological diseases.^6^, ^7^, ^8^, ^9^, ^10^ For instance: JKAP inhibits T‐cell receptor signaling and autoimmunity via regulating Lck and MAP4K family kinases^8^, ^11^; meanwhile, it represses CD4^+^ T‐cell differentiation into Th1 and Th17 cells in systemic lupus erythematosus nephritis and inflammatory bowel disease^12^, ^13^; Furthermore, it modifies PKA‐dependent phosphorylated TAU and CREB activation in Alzheimer disease, and is aberrant in schizophrenia.^9^, ^10^ Aside from these, it is also reported that CD4^+^ T cells are implicated in PD overall progression and especially cognitive impairment.^14^, ^15^ To sum up, we speculated that JKAP may be implicated in PD via its interaction with CD4^+^ T cells.

Therefore, the current study aimed to investigate the correlation of JKAP with Th1/Th2/Th17 cells and their clinical roles in PD patients, and then further explore the effect of JKAP on regulating CD4^+^ T‐cell differentiation in PD.

## Methods

### Subjects

From January 2018 to June 2020, this study consecutively recruited 50 de novo PD patients treated in our hospital. All eligible patients were older than 18 years and met the clinical diagnostic criteria for PD established by movement disorder society.^16^ Before enrollment, patients’ medical history and current complications were examined carefully. If patients had a history or were currently complicated with other neurodegenerative diseases, nervous system diseases, autoimmune diseases, hematologic malignancies, or cancers, they were excluded from the study. In addition to the PD patients, this study also enrolled 50 healthy subjects as controls. All controls were enrolled during physical examinations. On the enrollment, in order to match the age and sex of the patients with the controls, the age of controls was limited between 55 and 75 years old, and the gender ratio (male vs. female) was set as 2:1. The medical histories of controls were carefully checked and confirmed that they had no history of neurodegenerative diseases, nervous system diseases, autoimmune diseases, hematologic malignancies, or cancers. The written informed consents were acquired from all subjects, and received the ethical approval from the Institutional Review Board of Zhongshan Hospital Xiamen University.

### Clinical assessment for PD patients

Demographic information of PD patients was documented after recruitment. The main clinical symptom and severity of PD were evaluated using Unified Parkinson’s Disease Rating Scale (UPDRS), which comprised of four parts^17^: UPDRS‐I: non‐motor experiences of daily living; UPDRS‐II: motor experiences of daily living; UPDRS‐III: motor examination; UPDRS‐IV: motor complications. The score of each part in the UPDRS was calculated, respectively. Moreover, cognitive impairment of PD was also assessed using Mini‐Mental State Examination (MMSE) scale. Patients were considered cognitively impaired if their MMSE score was less than 27.^18^

### Sample collection and determination

Whole blood samples were collected from PD patients and controls. Then serum and peripheral blood mononuclear cell (PBMC) were isolated from whole blood by gradient density centrifugation with the use of Ficoll^®^ PM 400 (Sigma, USA). The JKAP concentration in serum samples was tested using Human JKAP Enzyme‐Linked Immunosorbent Assay (ELISA) Kit (Shanghai Enzyme‐linked Biotechnology Co., Ltd, China), which was performed following the instructions provided by the manufacturer. After the isolation of PBMC, the CD4 positive (CD4^+^) T cells were separated by Dynabeads™ Untouched™ Human CD4 T Cells Kit (Invitrogen, USA) in accordance with the kit’s instructions. Then Th1, Th2, and Th17 cell proportion in the CD4^+^ T cells was determined by flow cytometric analysis using Human Th1/Th2/Th17 Phenotyping Kit (BD Company, USA), which was performed referring to the experiment protocol of the kit.

### Lentivirus transduction

The CD4^+^ T cells used in in vitro experiments are derived from a same patient: the patient was a male with 67 years old, who had UPDRS‐I/II/III/IV scores with 3, 14, 18, and 6, respectively; meanwhile, he had a MMSE score of 28. The *Lentivirus* including JKAP overexpressing (OE), JKAP knocking‐down (KD), and negative control (NC) *Lentivirus* were provided by Shanghai HanBio Co., Ltd. (Shanghai, China). For the transduction, the CD4^+^ T cells (obtained from PBMC of PD patients) were preincubated with 5 μg/mL CD3 antibody (eBioscience, USA) and 2 μg/mL CD28 antibody (eBioscience, USA) for 12 hours. Then, the CD4^+^ T cells were infected with *Lentivirus* at a multiplicity of infection (MOI) of 100 overnight, and 6 μg/mL polybrene (Sigma, USA) was in present. Subsequently, the transduced CD4^+^ T cells were cultured with 5 μg/mL CD3 antibody (eBioscience, USA) and 2 μg/mL CD28 antibody (eBioscience, USA) for another 72 hours. The CD4^+^ T cells without *Lentivirus* infection were set as blank control.

### Reverse transcription quantitative polymerase chain reaction (RT‐qPCR)

The cells were collected after *Lentivirus* transduction, and the total RNA extraction was completed with TRIzol™ Reagent (Invitrogen, USA). The reverse transcription and qPCR were conducted with QuantiNova Reverse Transcription Kit (Qiagen, Germany) and QuantiNova SYBR Green PCR Kit (Qiagen, Germany), respectively. For calculating the result, the 2^‐ΔΔCt^ method was applied. The primers (5′ ‐> 3′) of JKAP and β‐actin were as follow: JKAP forward, GCCAGGCCTATGTTGGAGGGAGTT, JKAP reverse, TGTATGCGATCACCAGTGTCAC; β‐actin forward, TCGTGCGTGACATTAAGGAGAAG, β‐actin reverse, AGGAAGGAAGGCTGGAAGAGTG.

### Western blot

To isolate and quantify the total protein, the RIPA Lysis Buffer (Beyotime, China) and Enhanced BCA Protein Assay Kit (Beyotime, China) were adopted, respectively. Then, the sodium dodecyl sulfate polyacrylamide gel electrophoresis was carried out with 4%–20% precast gel (Willget, China). Subsequently, the protein was transferred to polyvinylidene fluoride membrane (PALL, Germany), followed by incubating with JKAP polyclonal antibody (1: 1500) (Invitrogen, USA) and HRP‐conjugated goat anti‐rabbit IgG (H + L) secondary antibody (1:50,000), sequentially. At last, BeyoECL Star (Beyotime, China) was used to visualize the protein bands.

### CD4^+^ T‐cell activation

CD25 and CD69 are two commonly used markers for evaluating CD4^+^ T‐cell activation according to previous studies,^12^, ^19^ which were further detected in our experiments. In brief, the cells were harvested, and stained with FITC‐conjugated CD25 rat mAb (1:20) (eBioscience, USA) or FITC‐conjugated CD69 mouse mAb (1:20) (eBioscience, USA). Then, the proportion of CD25‐positive (CD25^+^) or CD69‐positive (CD69^+^) cells was analyzed with a flow cytometer (BD, USA).

### Enzyme‐linked immunosorbent assay (ELISA)

The cell supernatant was collected, and then the level of IFN‐γ, IL‐10, and IL‐17 was measured using IFN‐γ Human ELISA Kit (Invitrogen, USA), IL‐10 Human ELISA Kit (Invitrogen, USA), and IL‐17 Human ELISA Kit (Invitrogen, USA), accordingly. The measurement was completed following the kits’ protocols.

### Statistical analysis

Comparisons between two groups were analyzed by Wilcoxon rank sum test. Multiple comparisons were analyzed by Tukey’s test. Correlation between two variables was analyzed by Spearman’s rank correlation test. The performance of variable in differentiating PD patients from controls was estimated using the receiver‐operating characteristic (ROC) curve and the area under the ROC curve (AUC). SPSS 22.0 (IBM, USA) and GraphPad Prism 7.01 (GraphPad Software Inc., USA) were applied to complete the data analysis and graph construction. Statistical significance was set as *P* value <0.05. In the in vitro experiment graphs, *P* value <0.05, <0.01, and <0.001 were labeled as *, **, and ***, respectively; non‐statistical significance was labeled as NS.

## Results

### Characteristics of PD patients

The enrolled PD patients had an age of 67.1 ± 5.6 years and included 35 (70.0%) males /15 (30.0%) females (Table 1). UPDRS‐I/II/III/IV scores were 3.4 ± 1.3, 9.7 ± 4.1, 14.6 ± 5.3, and 5.3 ± 2.0, respectively. Besides, MMSE score was 27.3 ± 1.5, with 10 (20.0%) patients having cognitive impairment defined by MMSE score less than 27.

**Table 1:** PD patients’ characteristics.

Items	PD patients (N = 50)
Age (years), mean±SD	67.1 ± 5.6
Gender, No. (%)	
Female	15 (30.0)
Male	35 (70.0)
UPDRS‐I, mean ± SD	3.4 ± 1.3
UPDRS‐II, mean ± SD	9.7 ± 4.1
UPDRS‐III, mean ± SD	14.6 ± 5.3
UPDRS‐IV, mean ± SD	5.3 ± 2.0
MMSE score, mean ± SD	27.3 ± 1.5
Cognitive impairment, No. (%)	
No	40 (80.0)
Yes	10 (20.0)

MMSE, mini‐mental state examination; PD, Parkinson's disease; SD, standard deviation; UPDRS, the unified Parkinson's disease rating scale.

### JKAP, Th1, Th2, and Th17 levels in PD patients and controls

**Figure 1:**
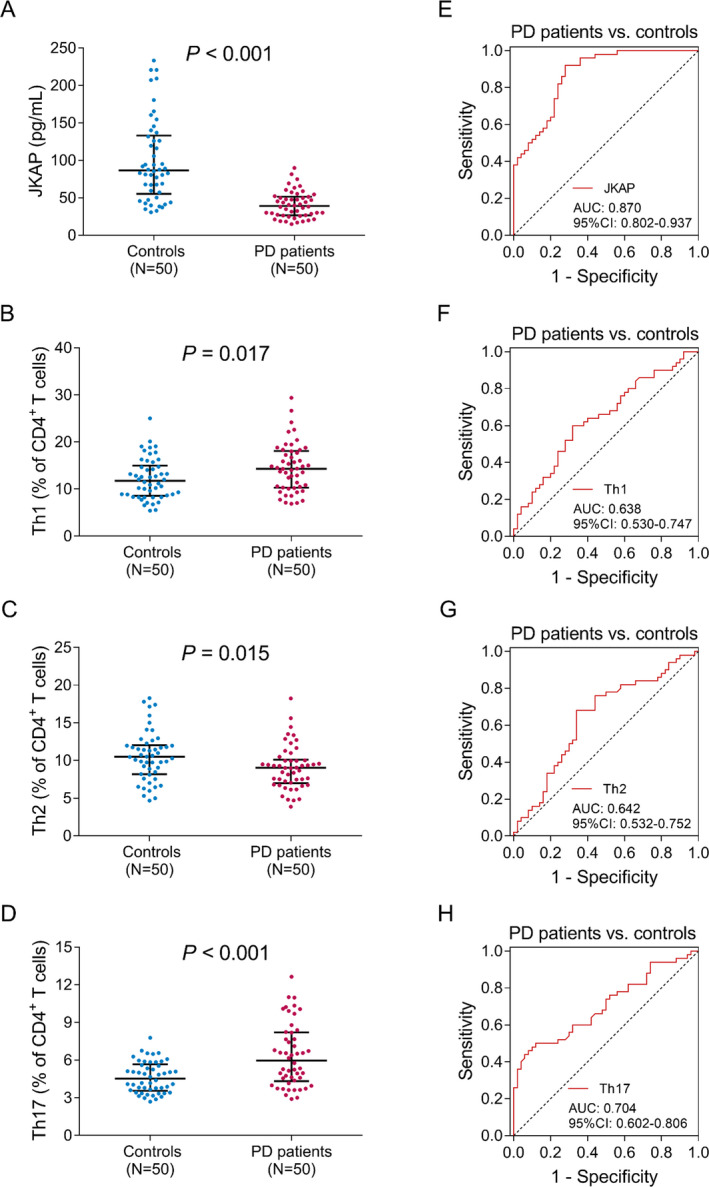
Comparison of JKAP, Th1, Th2, and Th17 cells. Comparison of JKAP (A), Th1 (B), Th2 (C), and Th17 (D) cells between PD patients and controls. ROC curve analyses of JKAP (E), Th1 (F), Th2 (G), and Th17 (H) cells for distinguishing PD patients from controls. JKAP, JNK pathway‐associated phosphatase; Th, T helper; ROC, receiver‐operating characteristic; PD, Parkinson's disease.

JKAP was greatly decreased in PD patients compared to controls (*P* < 0.001) (Fig. 1A). Besides, Th1 (*P* = 0.017) and Th17 (*P* < 0.001) cells were higher, while Th2 (*P* = 0.015) cells were lower in PD patients compared with controls (Fig. 1B‐D). Subsequent ROC curve analyses discovered that JKAP showed a good value for telling PD patients from controls (AUC: 0.870, 95% CI: 0.802‐0.937); Th17 cells disclosed an acceptable value for distinguishing PD patients from controls (AUC: 0.704, 95% CI: 0.602‐0.806); while Th1 and Th2 cells only revealed fair values for differentiating PD patients from controls (Th1‐AUC: 0.638, 95% CI: 0.530‐0.747; Th2‐ AUC: 0.642, 95% CI: 0.532‐0.752) (Fig. 1E‐H). As to Th1/Th2 ratio, it was increased in PD patients compared to controls (*P* = 0004), which also showed some potent to distinguish PD patients from controls (AUC: 0.667, 95% CI: 0.561‐0.774) (Fig. S1A‐B).

### Correlation of JKAP with Th1, Th2, and Th17 cells in PD patients and controls

**Figure 2:**
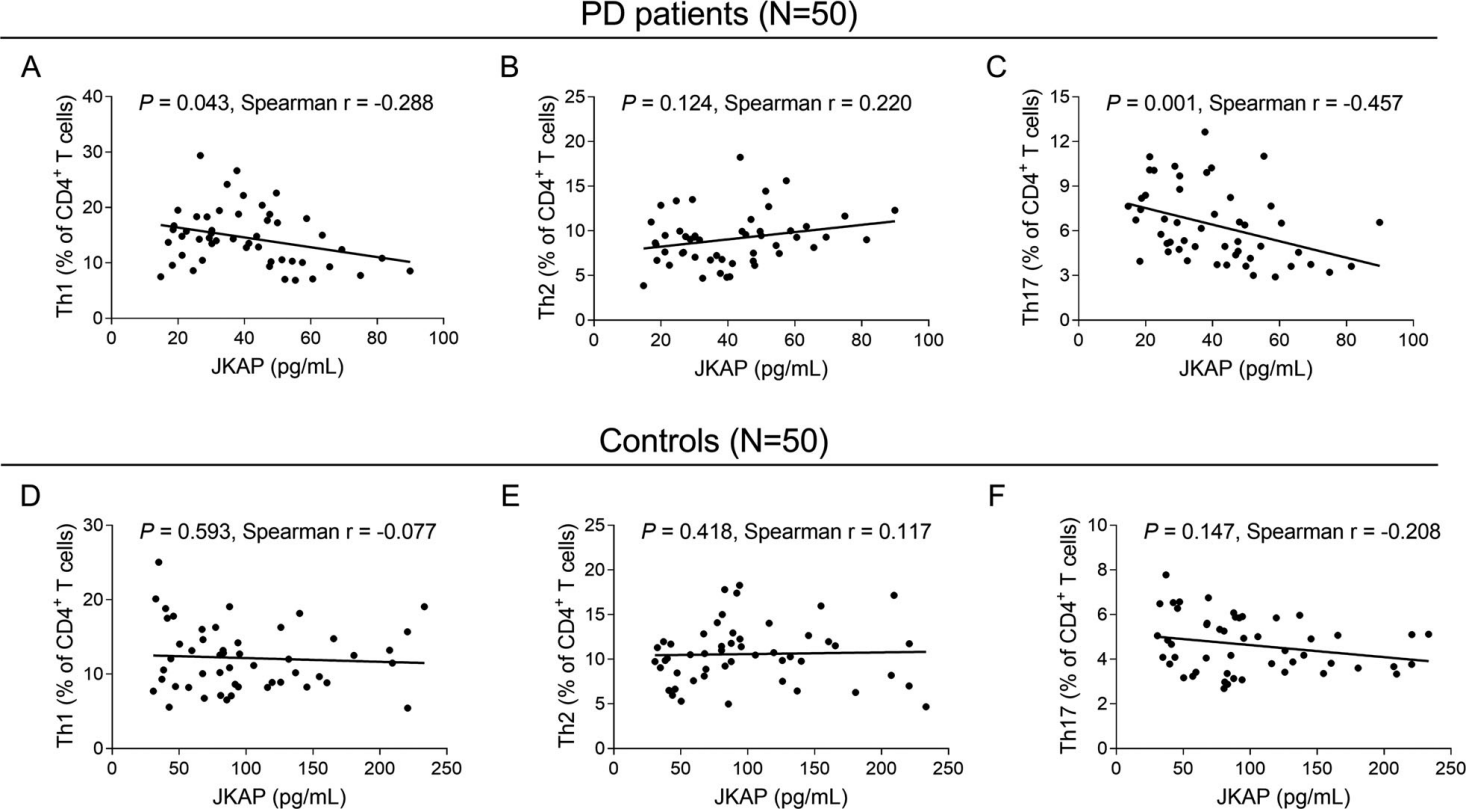
Correlation between JKAP and Th1, Th2, Th17 cells. Correlation of JKAP with Th1 (A), Th2 (B), and Th17 (C) cells in PD patients. Correlation of JKAP with Th1 (D), Th2 (E), and Th17 (F) cells in controls. JKAP, JNK pathway‐associated phosphatase; Th, T helper; PD, Parkinson's disease.

In PD patients, JKAP was negatively correlated with Th1 cells (*P* = 0.043, r = −0.288) and Th17 cells (*P* = 0.001, r = −0.457), while did not associate with Th2 cells (*P* = 0.124, r = 0.220) (Fig. 2A‐C). In controls, JKAP was not correlated with Th1 cells (*P* = 0.593, r = −0.077), Th2 cells (*P* = 0.418, r = 0.117), or Th17 cells (*P* = 0.147, r = −0.208) (Fig. 2D‐F). As to Th1/Th2 ratio, JKAP was negatively correlated with Th1/Th2 ratio in PD patients (*P* = 0.029, r = −0.309) but not in controls (*P* = 0.684, r = 0.059) (Fig. S1C‐D).

### Correlation of JKAP with UPDRS and MMSE scores in PD patients

**Figure 3:**
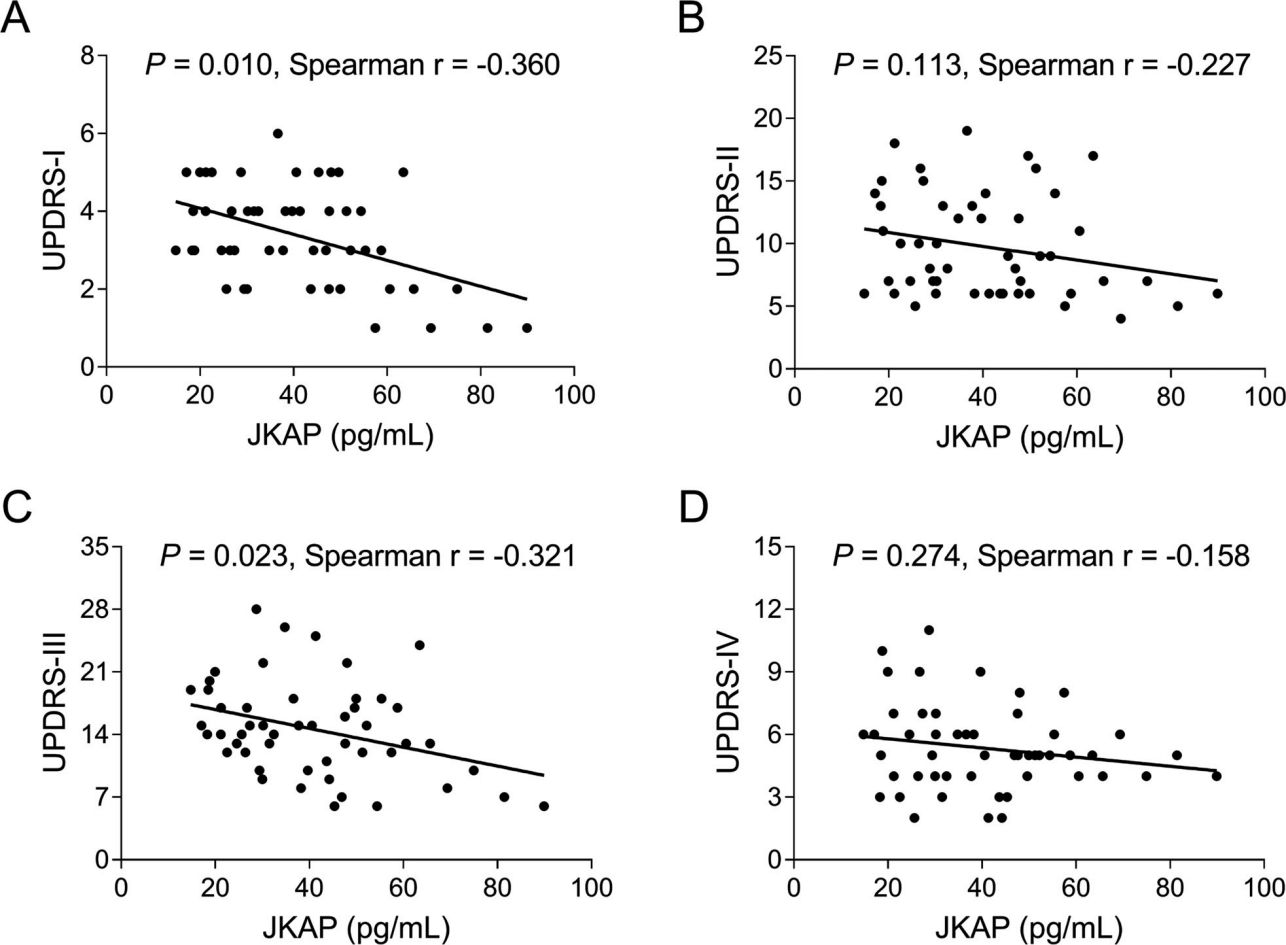
Correlation between JKAP and UPDRS scores. Correlation of JKAP with UPDRS‐I score (A), UPDRS‐II score (B), UPDRS‐III score (C) and UPDRS‐IV score (D) in PD patients. JKAP, JNK pathway‐associated phosphatase; UPDRS, unified Parkinson’s disease rating scale; PD, Parkinson's disease.

**Figure 4:**
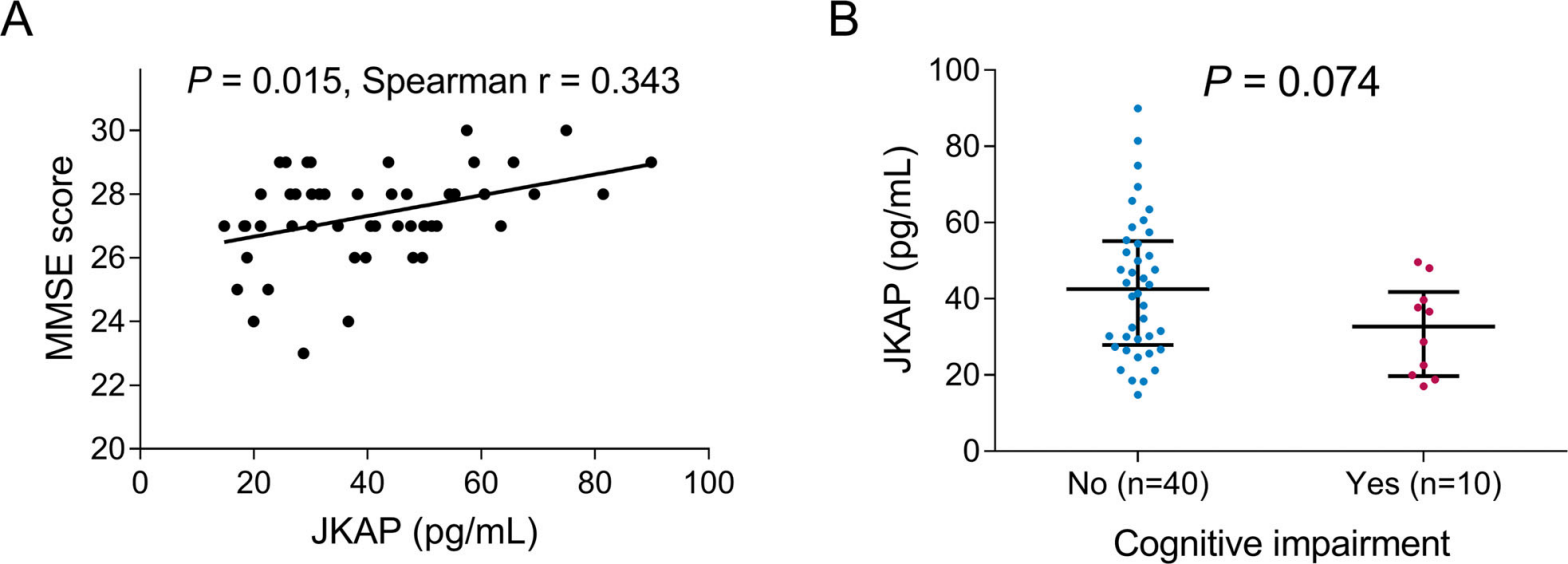
Correlation between JKAP and MMSE score. Correlation of JKAP with MMSE score (A) and cognitive impairment defined as MMSE score less than 27 (B) in PD patients. JKAP, JNK pathway‐associated phosphatase; MMSE, mini‐mental state examination; PD, Parkinson's disease.

JKAP was negatively correlated with some parts of UPDRS. In detail, JKAP was negatively correlated with UPDRS‐I score (*P* = 0.010, r = −0.360) and UPDRS‐III score (*P* = 0.023, r = −0.321), while was not related to UPDRS‐II score (*P* = 0.113, r = −0.227) or UPDRS‐IV score (*P* = 0.274, r = −0.158) (Fig. 3A‐D). In terms of MMSE score, JKAP was positively correlated with MMSE score (*P* = 0.015, r = 0.343) (Fig. 4A); while it only showed a trend of decrease in cognitive impairment patients compared to non‐cognitive impairment patients, which was not statistically significant (*P* = 0.074) (Fig. 4B).

### Correlation of Th1/Th2/Th17 cells with UPDRS and MMSE scores in PD patients

Th1 cells positively correlated with UPDRS‐I score (*P* = 0.009, r = 0.368) while negatively correlated with MMSE score (*P* = 0.008, r = −0.373). Th2 cells only positively related to MMSE score (*P* = 0.002, r = 0.435). Th17 cells positively associated with UPDRS‐I score (*P* = 0.011, r = 0.356) and UPDRS‐IV score (*P* = 0.022, r = 0.324), but negatively associated with MMSE score (*P* = 0.027, r = −0.312). The more detailed information about the correlation of Th1/Th2/Th17 cells with UPDRS and MMSE scores was shown in Table 2.

**Table 2:** Correlation of Th1, Th2, and Th17 cell proportion with UPDRS and MMSE score.

Items	Th1 cell		Th2 cell		Th17 cell	
	Spearman r	*P* value	Spearman r	*P* value	Spearman r	*P* value
UPDRS‐I	0.368	0.009	−0.264	0.064	0.356	0.011
UPDRS‐II	0.142	0.325	−0.145	0.313	0.176	0.222
UPDRS‐III	0.135	0.350	−0.273	0.056	0.164	0.256
UPDRS‐IV	0.062	0.669	−0.159	0.271	0.324	0.022
MMSE score	−0.373	0.008	0.435	0.002	−0.312	0.027

Correlation was determined by Spearman’s rank correlation test. MMSE, mini‐mental state examination; UPDRS, the unified Parkinson's disease rating scale.

### JKAP inhibited CD4^+^ T‐cell activation and Th1, Th17 differentiation in PD

**Figure 5:**
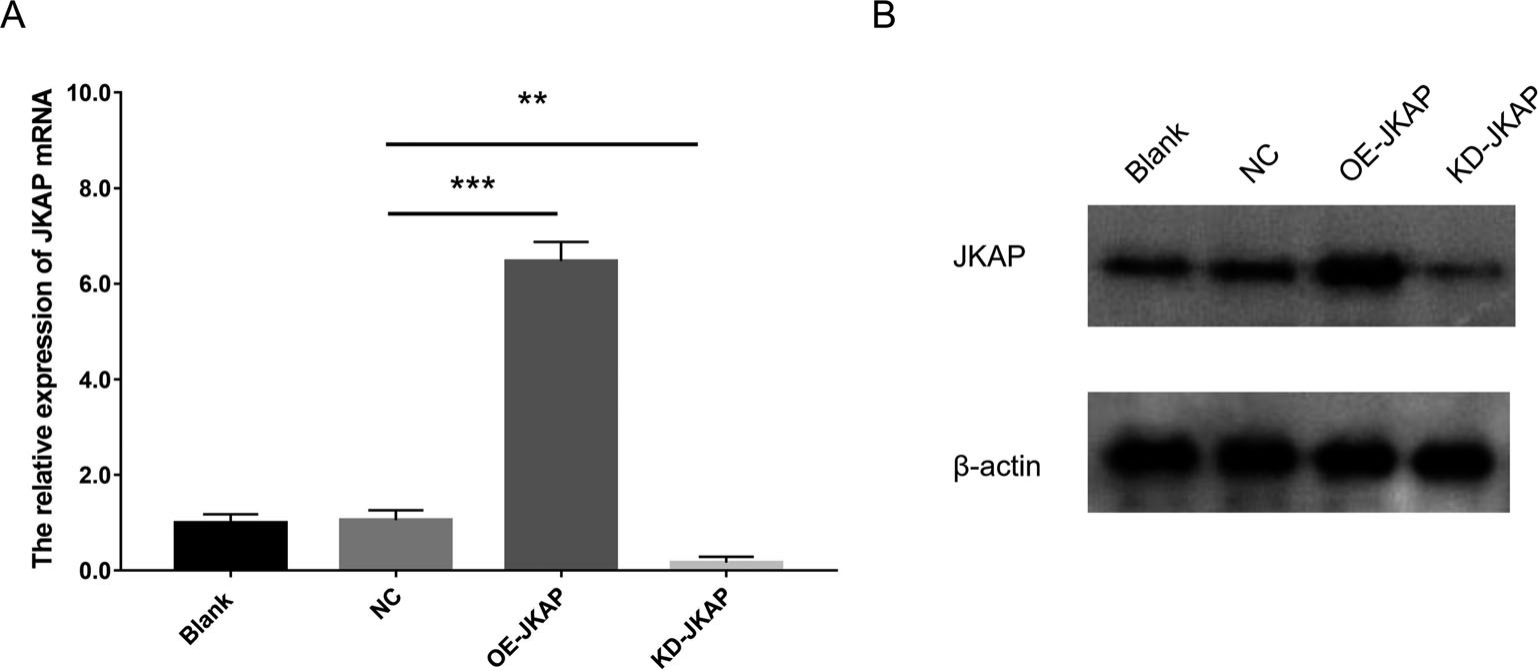
JKAP expression after transfection. JKAP mRNA (A) and protein (B) expression among Blank, NC, OE‐JKAP, and KD‐JKAP groups in CD4^+^ T‐cells isolated from PD patients. JKAP, JNK pathway‐associated phosphatase; NC, negative control; OE, overexpression; KD, knockdown; PD, Parkinson's disease. **P < 0.01, *** *P* < 0.001.

**Figure 6:**
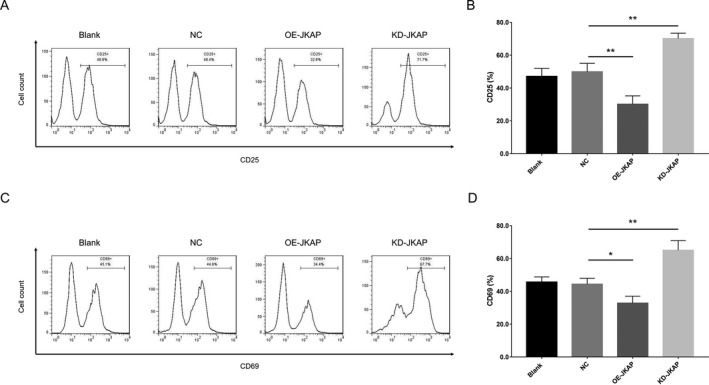
CD4^+^ T‐cell inactivation after transfection. CD25^+^ cell proportion (A), (B) and CD69^+^ cell proportion (C), (D) among Blank, NC, OE‐JKAP, and KD‐JKAP groups in CD4^+^ T‐cells isolated from PD patients. JKAP, JNK pathway‐associated phosphatase; NC, negative control; OE, overexpression; KD, knockdown; PD, Parkinson's disease. **P < 0.01, *** *P* < 0.001.

**Figure 7:**
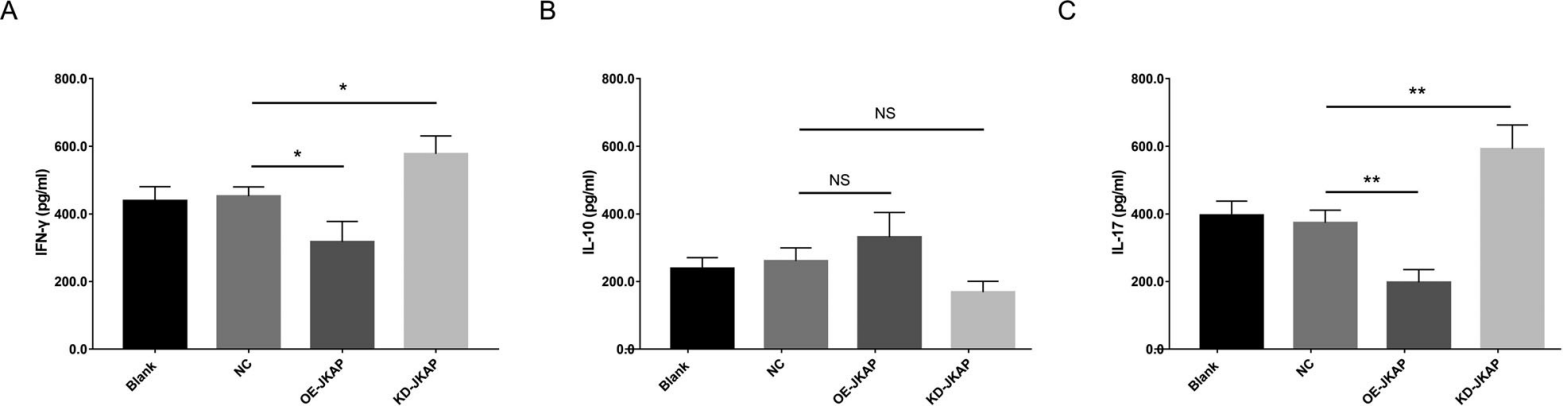
IFN‐γ, IL‐10, and IL‐17 expressions after transfection. IFN‐γ (Th1 secreted cytokine) (A), IL‐10 (Th2 secreted cytokine) (B) and IL‐17 (Th17 secreted cytokine) (C) expressions among Blank, NC, OE‐JKAP, and KD‐JKAP groups in CD4^+^ T‐cells isolated from PD patients. IFN‐γ, interferon‐γ; IL‐10, interleukin 10; IL‐17, interleukin 17; Th, T helper; JKAP, JNK pathway‐associated phosphatase; NC, negative control; OE, overexpression; KD, knockdown; PD, Parkinson's disease.

After transfection, JKAP expression was enhanced in the OE‐JKAP group, while repressed in the KD‐JKAP group, compared to the NC group, suggesting good transfecting efficiency (Fig. 5A‐B). Subsequent experiments observed that CD25^+^ cell percentage and CD69^+^ cell percentage were both decreased in the OE‐JKAP group, while increased in the KD‐JKAP group, compared to the NC group, implying JKAP inhibited CD4^+^ T‐cell activation (Fig. 6A‐D). Furthermore, Th1 secreted cytokine (IFN‐γ) and Th17 secreted cytokine (IL‐17) were both reduced in the OE‐JKAP group, while elevated in the KD‐JKAP group, compared to the NC group; while Th2 secreted cytokine (IL‐10) did not differ among the groups (Fig. 7A‐C); implying JKAP inhibited Th1 and Th17 differentiation in PD.

## Discussion

JKAP, as one key member of DUSP family, is reported to possess clinical value reflecting disease risk and severity of several immune/inflammatory‐related disease and neurological diseases. For instance, a study uncovers that serum JKAP is downregulated in rheumatoid arthritis patients compared to controls, and it is negatively correlated erythrocyte sedimentation rate, C‐reactive protein and disease activity in RA patients^6^; another study discloses that blood JKAP reduction correlates with asthmatic exacerbation and pro‐inflammatory cytokines in asthmatic children^7^; furthermore, some reports discovers that JKAP hypermethylation exists and correlates with TAU phosphorylation in AD patients, as well as is involved in the interaction between utero famine exposure and schizophrenia.^9^, ^10^

However, as the clinical role of JKAP in PD patients, no report has been revealed. Therefore, we performed the current study and observed that serum JKAP was greatly decreased in PD patients compared to controls, correlates with lower UPDRS‐I score, UPDRS‐II score while higher MMSE score in PD patients. The possible explanations include: (1) JKAP insufficiency decreased neuro viability and function via its hypermethylation and inflammation regulation; therefore, it was downregulated in PD patients compared to controls, and correlated with some part of UPDRS scores^6^, ^7^, ^8^, ^9^, ^10^; (2) JKAP inactivated T‐cell signaling and interacted with Th1 as well as Th17, resulting in aberrant immune environment and inflammation to affect PD progression; therefore, it correlated with some part of UPDRS scores and MMSE score^8^, ^11^, ^12^, ^13^, ^20^, ^21^; (3) JKAP repressed Th17 cell differentiation, the latter was closely involved in the cognitive impairment in neurological diseases including PD; therefore, JKAP correlated with MMSE score.^22^

In terms of regulation of CD4^+^ T cells, JKAP is reported to repress CD4^+^ T‐cell activation as well as its differentiation into Th1 and Th17 cells in systemic lupus erythematosus nephritis and inflammatory bowel disease.^12^, ^13^ Clinically, JKAP negatively correlates with Th17 cell secreted IL‐17 in inflammatory bowel disease patients, and negatively correlates with Th1 cells as well as Th17 cells in sepsis patients.^12^, ^23^ But to date, the relation between JKAP and CD4^+^ T cells in PD patients is not uncovered yet. Our study observed JKAP was negatively correlated with Th1 and Th17 cells, while was not related to Th2 cells in PD patients; furthermore, we performed in vitro experiments based on CD4^+^ T cells isolated from PD patients, and discovered that JKAP inactivated CD4^+^ T cells as well as inhibited their differentiation into Th1 and Th17 cells, which further validated the clinical findings. The following items might explain these findings: (1) JKAP dephosphorylated tyrosine‐394 residue to inactivate Lck, further obstructed T‐cell receptor signaling, then regulated CD4^+^ T‐cell activation and differentiation^11^; (2) JKAP interacted with MAP4K to regulate CD4^+^ T‐cell activation and differentiation.^8^

With regard to the implication of Th1, Th2, and Th17 cells in PD, accumulating studies have been reported. A study finds that Th1/Th2 ratio and Th17/Treg ratios are greatly enhanced in PD patients compared to controls^24^; another interesting study observes that Th1‐biased immune signature is not only presented in drug‐naive patients but also in dopaminergic drugs treated patients^25^; furthermore, Th1, Th2, and Th17 cells correlates with cognitive impairment to some extent in PD patients especially drug naive PD patients.^22^ These indicated Th1, Th2, and Th17 are related to PD. In this study, we also observed that Th1 and Th17 correlated with some parts of UPDRS scores and MMSE score, while Th1 only correlated with MMSE score. These findings were in‐line with previous studies.

Several limitations of this study needed to be clarified: (1) The samples size was relatively small that reduced the statistical power and weakened the possibility of subgroup analyses; therefore, large sample size subjects could be enrolled to validate the findings; furthermore, future enrollment of a validation cohort was also needed. (2) Only blood samples were acquired from subjects, while cerebrospinal fluid samples were not included, which needed further exploration; (3) The JKAP expression in PD patients was detected once instead of multiple measurement during disease course and/or treatment; therefore, the disease monitor value of JKAP needed further investigation.

## Conclusions

In conclusion, JKAP associates with disease risk and severity, correlates with Th1 and Th17 cells, and regulates CD4^+^ T‐cell activation/differentiation in PD.

## Funding Information

This study was supported by Medical Elite Cultivation Program of Fujian, China (No. 2018‐ZQN‐85).

## Conflicts of Interest

The authors of this work have nothing to disclose.

## Supporting information

**Supplementary Figure S1**. Th1/Th2 ratio. Comparison of Th1/Th2 ratio between PD patients and controls (A). ROC curve analysis of Th1/Th2 ratio for distinguishing PD patients from controls (B). Correlation of JKAP with Th1/Th2 ratio in PD patients (C) and controls (D). JKAP, JNK pathway‐associated phosphatase; Th, T helper; ROC, receiver‐operating characteristic; PD, Parkinson's disease.
