# Supplementary figures and images for: JKAP relates to disease risk, severity, and Th1 and Th17 differentiation in Parkinson's disease
Source: Ann Clin Transl Neurol. 2021 Jul 21;8(9):1786–95. doi: 10.1002/acn3.51420 (PMC8419400; doi:10.1002/acn3.51420)

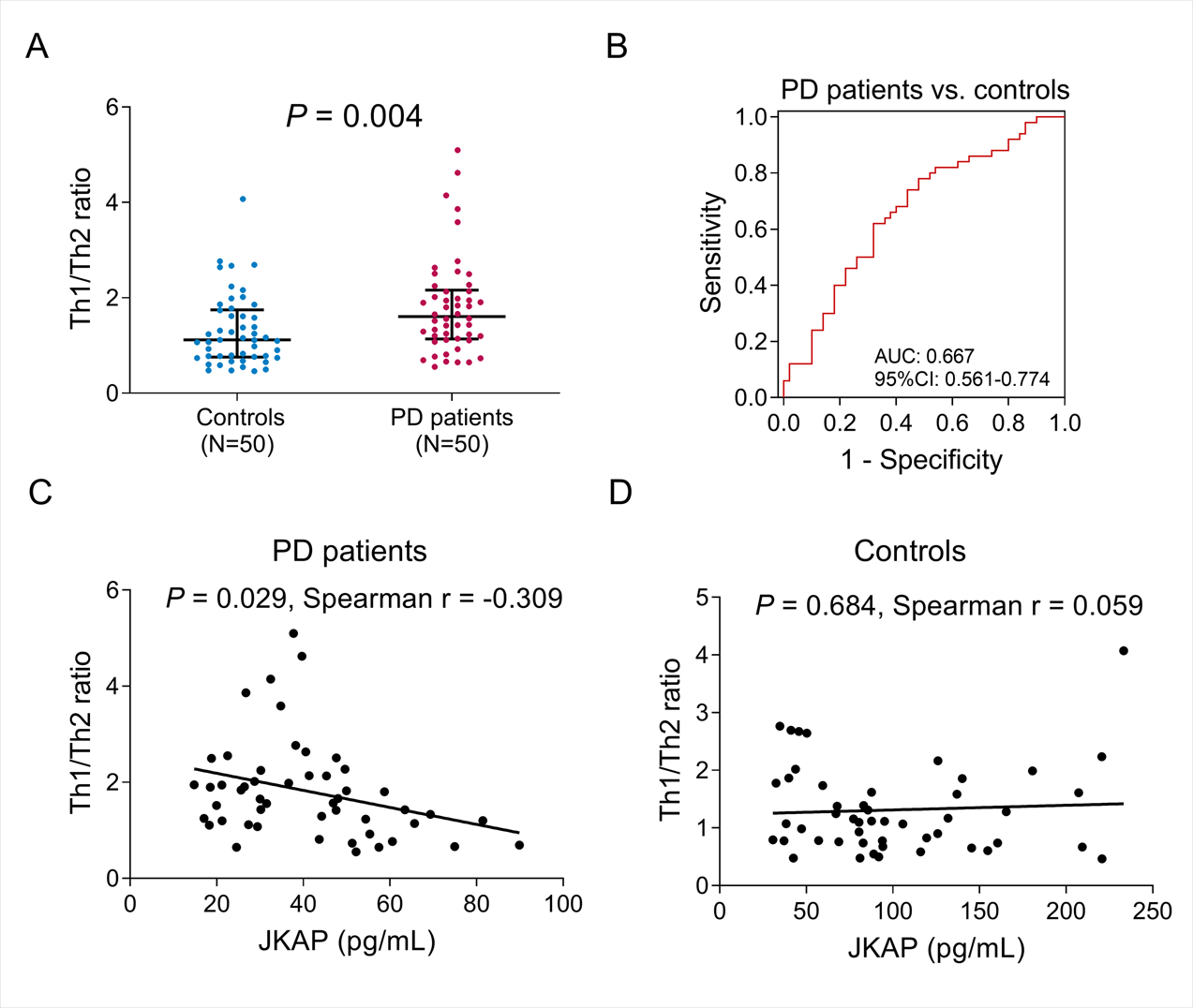

Supplement: Supplementary file 1 — Supplementary Figure S1. Th1/Th2 ratio. Comparison of Th1/Th2 ratio between PD patients and controls (A). ROC curve analysis of Th1/Th2 ratio for distinguishing PD patients from controls (B). Correlation of JKAP with Th1/Th2 ratio in PD patients (C) and controls (D). JKAP, JNK pathway‐associated phosphatase; Th, T helper; ROC, receiver‐operating characteristic; PD, Parkinson's disease. [file ACN3-8-1786-s001.tif]
